# Bacmethy: A novel and convenient tool for investigating bacterial DNA methylation pattern and their transcriptional regulation effects

**DOI:** 10.1002/imt2.186

**Published:** 2024-03-19

**Authors:** Ji‐Hong Liu, Yizhou Zhang, Ning Zhou, Jiale He, Jing Xu, Zhao Cai, Liang Yang, Yang Liu

**Affiliations:** ^1^ Medical Research Center Southern University of Science and Technology Hospital Shenzhen China; ^2^ School of Medicine, Key University Laboratory of Metabolism and Health of Guangdong Southern University of Science and Technology Shenzhen China; ^3^ Clinical Laboratory Southern University of Science and Technology Hospital Shenzhen China; ^4^ Shenzhen Third People's Hospital, The Second Affiliated Hospital of Southern University of Science and Technology National Clinical Research Center for Infectious Disease Shenzhen China

**Keywords:** bacteria, DNA methylation, SMRT‐seq, transcriptional regulation, undermethylation

## Abstract

DNA methylation serves as the primary mode of epigenetic regulation in prokaryotes, particularly through transcriptional regulation. With the rapid implementation of third‐generation sequencing technology, we are currently experiencing a golden age of bacterial epigenomics. However, there has been a lack of comprehensive research exploring the versatility and consequential impact of bacterial DNA methylome on cellular and physiological functions. There is a critical need for a user‐friendly bioinformatics tool that can effectively characterize DNA methylation modification features and predict the regulation patterns. To address this gap, the current study introduces Bacmethy, an innovative tool that utilizes SMRT‐seq data and offers a range of analytical modules. First, the tool classifies methylation sites in the genome, highlighting the distinct regulations present under varying modification fractions and location enrichment. Furthermore, this tool enables us to identify regulatory region methylation and potential *cis* and *trans* interactions between methylation sites and regulatory effectors. Using benchmark data sets and our data, we show that our tool facilitates the understanding of the distinctive traits of DNA methylation modifications and predicts transcriptional regulation effects on important physiological and pathological functions. Bacmethy code is freely available, and the Docker image is downloadable. Bacmethy has been made available as a user‐friendly web server interface at https://bacmethy.med.sustech.edu.cn.

## Highlights

- Bacmethy tool provides a one‐stop analysis and visualization pipeline for effectively characterizing bacterial DNA methylation modification features and predicting the regulation patterns.
- Bacmethy offers both a local run function and an online interface analysis service, providing significant convenience for researchers without coding abilities.
- Bacmethy provides useful information for decoding the underlying molecular mechanisms of how DNA methylation regulates bacterial cellular and physiological functions.

## Graphical abstract

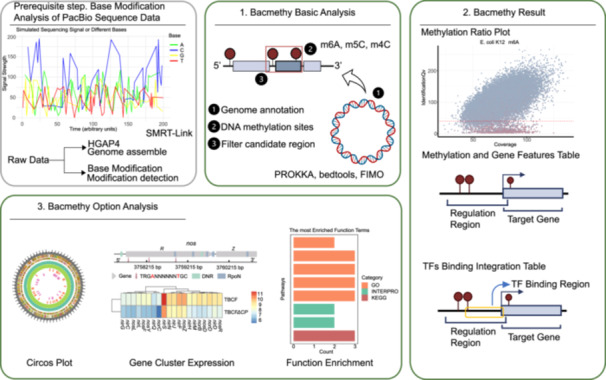

## INTRODUCTION

In prokaryotes, epigenetic control mainly relies on DNA methylation, which is mediated by DNA methyltransferase (DNA MTase) [1, 2]. DNA MTases transfer a methyl group from the donor S‐adenosyl‐l‐methionine to specific locations on the target bases, forming different forms of modifications, mainly including N6‐methyladenine (m6A, the most common type in bacteria), N4‐methylcytosine (m4C), and 5‐methylcytosine (m5C) [3, 4].

DNA methylation affects transcription through different molecular mechanisms. Methylated bases protrude into the main groove of the DNA double helix, thereby affecting the interaction of proteins, either negatively or positively [5, 6]. Notably, under certain conditions, protein binding to DNA can prevent methylation, resulting in hypomethylation or undermethylation, characterized by low levels of methylation [7, 8]. Methylation modification may also affect the topological structure of chromosomes [9, 10]. Characterizing methylome features can predict the impact of DNA methylation on transcriptional regulation and biological consequences [3, 11].

In the last few decades, there has been a surge in investigations of the versatility and functional effects of the bacterial DNA methylome, facilitated by the SMRT‐seq technology [2, 12, 13, 14]. SMRT‐seq is a powerful tool for detecting bacterial DNA methylation at a genome‐wide level, achieving single‐nucleotide resolution [3]. By 2023 December, this technology has been used to generate the majority of >5000 mapped bacterial and archaea methylomes that are currently accessible in the centralized REBASE database, with even more undisclosed data [15].

SMRT‐seq data is in binary format and includes both raw sequence information and interpulse duration (IPD) signals. IPD signals, also known as the time intervals between pulses during sequencing, provide an indication of covalent modifications occurring within the template DNA [16]. Bioinformatic tools like SMRT‐Tool and SMRTLink are used for analyzing the SMRT‐seq raw data [17, 18]. The Base Modification Detection application can detect DNA methylation and construct motif‐specific models [17, 18]. However, there is a lack of convenient bioinformatics tool to systematically characterize DNA methylome features and predict transcriptional regulation effects based on SMRT‐seq data [14, 16].

To bridge this gap, we developed Bacmethy, an innovative tool for analyzing bacterial methylome. It features a classification system for methylation sites, scans for methylation/undermethylation positions, calculates enrichment significance of (under)methylated motifs in regulatory regions (RRs), and identifies nearby binding sites of transcription regulation effectors (transcription factors [TFs] or sigma factors). Bacmethy allows nonexpert users to conduct comprehensive bacterial methylome analysis. It is highly effective in facilitating a better understanding of DNA methylation modification and predicting transcriptional regulation.

## RESULTS

### Bacmethy pipeline implementation

**Figure 1:**
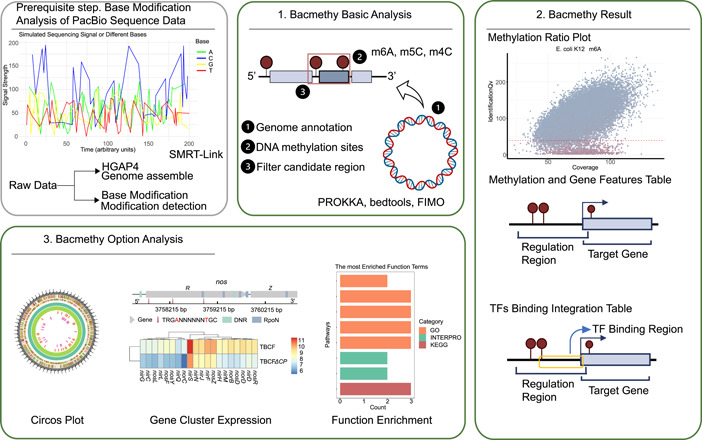
Bacmethy analysis workflow diagram. The pipeline includes prerequisite, basic, and option analysis. The results include “Methylation Ratio Plot,” “Methylation and Gene Features Table,” “TFs' Binding Integration Table,” “Circos plot of methylome,” “Function Enrichment,” and so on. TFs, transcription factors.

Users can run Bacmethy using a bash command or on the website (Figures 1, S1, and S2) with the requested information, including the name of the sample, modification detection files (motifs.csv and motifs.gff), and a complete genome sequence (fasta file). Among the customizable parameters, one is the threshold of methylation level, which offers users the option to utilize different thresholds of undermethylation and unmethylation modules included in the pipeline. Another customizable parameter is the TFs' binding module. Users should prepare the TF matrix and either place it under a designated directory in the pipeline or upload it to the web server.

To demonstrate the Bacmethy application workflow, the pipeline with default parameter settings was applied to analyze SMRT‐seq data summarized in Table 1. Considering the time efficiency of Bacmethy, the running time in the web server is correlated to methylation sites in each genome. For a 4000 methylation site strain (e.g., *Pseudomonas aeruginosa* TBCF), the running time of basic Bacmethy analysis will be less than 10 min.

**Table 1:** Bacterial strains with methylome data included in this study.

MTases investigated	Strain number	Species (abbreviation)	Predicted RM systems type	Orphan MTase	Recognize motif	Methylation type	References
M.Xsp91I	91	*Xanthobacter* species (*Xan*)	II	Y	G**A**NTC	m6A	[4]
M.CmaLM2III	LM2	*Clostridium mangenotii* (Cm)	I	N	GG**A**NNNNNVTAC	m6A	[4]
M.PaeZa2I	LYSZa2	*Pseudomonas aeruginosa* (*Pa*)	I	N	CCA**A**NNNNNNNCTC	m6A	This study
M.PaeZa7I	LYSZa7	*Pseudomonas aeruginosa* (*Pa*)	I	N	**A**GGNNNNNRTGT	m6A	[19]
M.PaeZa7II	LYSZa7	*Pseudomonas aeruginosa* (*Pa*)	III	Y	CCCG**A**G	m6A	[19]
M.PaeTBCFORFAP	TBCF	*Pseudomonas aeruginosa* (*Pa*)	I	N	RCC**A**NNNNNNNTGAR	m6A	[20]
M.PaeTBCFORFCP	TBCF	*Pseudomonas aeruginosa* (*Pa*)	I	Y	TRG**A**NNNNNNTGC	m6A	[20]
M.EcoKDam	MG1655	*Escherichia coli* (*E. coli*)	II	Y	G**A** TC	m6A	[4]
M.EcoKII	MG1655	*Escherichia coli* (E. coli)	II	Y	**C**CTGG	m4C	[4]
M.EcoKDcm	MG1655	*Escherichia coli* (*E. coli*)	II	N	C**C**WGG	m5C	[4]
M.EcoKI	MG1655	*Escherichia coli* (*E. coli*)	I	N	A**A**CNNNNNNGTGC	m6A	[4]
M.SauTCHI	USA300_TCH1516	*Staphylococcus aureus* (*Sa*)	I	N	AC**A**NNNNNNRTGG	m6A	[4]
M.SauTCHII	USA300_TCH1516	*Staphylococcus aureus* (*Sa*)	I	N	**A**GGNNNNNGAT	m6A	[4]

*Note*: This study includes the methylome data of 13 MTases from seven bacterial strains. The data of six MTases from the top four bacterial strains were obtained from GSE69872, while the SMRT‐seq data of the remaining three strains was generated in our laboratory (*Pseudomonas aeruginosa* TBCF, LYSZa2, and LYSZa7) [21]. These MTases are predicted to belong to different RM system types, and some of them are orphan MTases. The methylated nucleotide within the motif is highlighted in bold. The underlined letter denotes the nucleotide that pairs with the methylated nucleotide on the complementary strand.

Abbreviations: m6A, N6‐methyladenine; m4C, N4‐methylcytosine; m5C, 5‐methylcytosine; MTase, methyltransferase.

### Distribution of the methylated and un(der)methylated motifs

**Figure 2:**
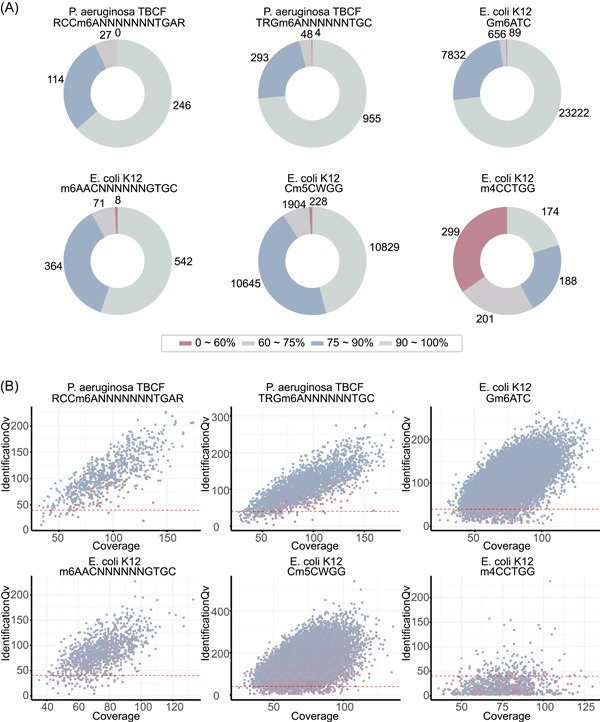
The distribution of methylation fraction and sequencing quality. (A) The distribution of methylation fraction per motif in the bacterial stains, where the title of each circle includes the strain name and recognition motif, and the counts indicate the number of motifs with diverse ratios of 0%−60% (red), 60%−75% (gray), 75%−90% (blue), and 90%−100% (green). The scatter plots for the quality distribution of methylated and un(der)methylated motifs across the strains per methyltransferase (MTase) motif (B) (six MTase motifs for *Pseudomonas aeruginosa* TBCF and *Escherichia coli* K12). The *x*‐axis shows the read coverage, and the *y*‐axis shows the identification QV. The methylation fraction is represented by dot color, with blue indicating a high methylation fraction and red indicating a low fraction. QV, quality value.

To investigate the precise methylome across bacterial genomes, we first identified the distribution of the three modification levels (methylation, undermethylation, or unmethylation) in different gene regions (regulatory or coding regions) per MTase motif in the bacterial genomes (Table S1). Our data demonstrate that there is significant diversity in the distribution of different MTase sites in the genomes. We further plot the distribution of methylation fraction per MTase motif for each strain (Figures 2A and S3A). For the majority of the MTase motifs in the bacterial strains examined, only a small proportion of low‐fraction methylation sites (methylation fraction <75%) were detected. However, there were four motifs (one motif in *Escherichia coli* K12, one in *Xm* LM2, and two motifs in *Staphylococcus aureus* USA300) where a significant number of low‐fraction methylation sites were observed. Next, we created the scatter plots for the quality distribution of methylated and un(der)methylated motifs across the strains per MTase motif (Figures 2B and S3B). The methylation feature of each site is represented by three variables (identification quality value (QV), methylation fraction, and reads coverage). The distribution of methylation fraction for each motif in the stains is quite diverse. Apart from unmethylated motif sites, there are varying numbers of undermethylated motif sites (with high identification QV and reads coverage, but low methylation fraction) in the strains. For example, *S. aureus* USA300 contains a very high level of undermethylated motif sites.

Additionally, we utilized Circos plots to visualize the distribution of DNA methylation sites in the tested strains. In the plot, the fifth ring (Figure S4; outer to inner) represents undermethylation and unmethylation motifs across the genome. The majority of motifs with methylation (Figure S4; fourth and sixth loops represent positive and negative strands, respectively, outer to inner) were randomly and evenly distributed throughout the genome.

**Figure 3:**
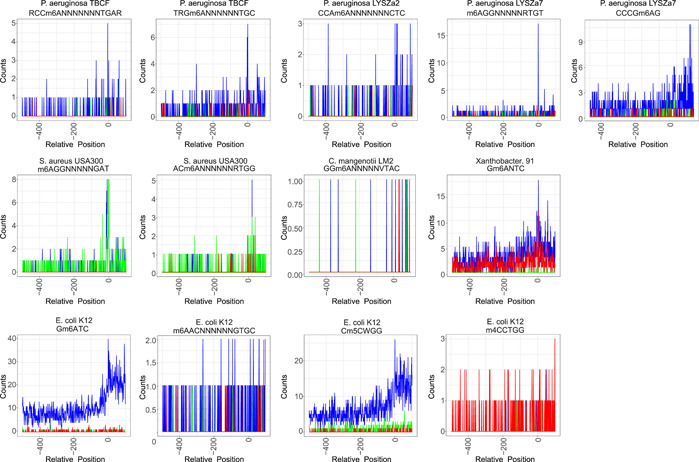
Number of methylated sites located at the regulation region for each methyltransferase recognition motif in different strains. The vertical axis shows the count of methylated (blue), unmethylated (red), or undermethylated (green) sites. The horizontal axis indicates the position of modification sites located around the ATG initiation codon.

To analyze the distribution of undermethylation and unmethylation sites in the regulation region, we generated location enrichment plots (Figure 3). These plots illustrated prominent peaks around the transcription start sites, suggesting a connection between the level of methylation modification and transcription start regulation. These dynamic changes in methylation levels could potentially be attributed to competition with DNA‐binding proteins.

### Functional enrichment of the methylated and un(der)methylated motifs

**Figure 4:**
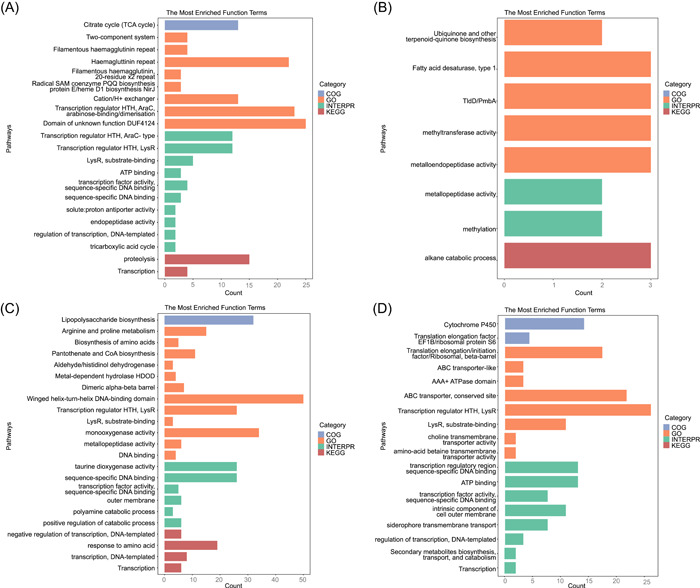
Function enrichment results of methylated genes in *Pseudomonas aeruginosa* strains. Methylated genes refer to genes with corresponding DNA methylation modification in transcriptional regulatory regions. The most enriched functional terms of genes associated with: (A) the TRGANNNNNNTGC motif sites in TBCF; (B) the RCCANNNNNNNTGAR motif sites in TBCF; (C) the CCCGAG motif sites in LYSZa7; (D) the AGGNNNNNRTGT motif sites in LYSZa7. COG category [20] and DAVID [22] tools were used for annotation.

To ascertain the potential functions of genes affected by DNA methylation, we conducted function enrichment analysis for DNA methylation‐related genes (referring to genes with corresponding DNA methylation modification in transcriptional RRs) in two *P. aeruginosa* strains (TBCF and LYSZa7). The most enriched functional terms are shown in Figure 4. The enrichment results for MTase motifs vary significantly among different strains. Notably, genes that encode transcription regulators and contain DNA‐binding domains were found to be enriched, indicating a regulatory role of methylation on transcriptional regulation.

### Scanning the gene regulation regions with methylated motifs to identify transcription regulation effector binding sites

**Figure 5:**
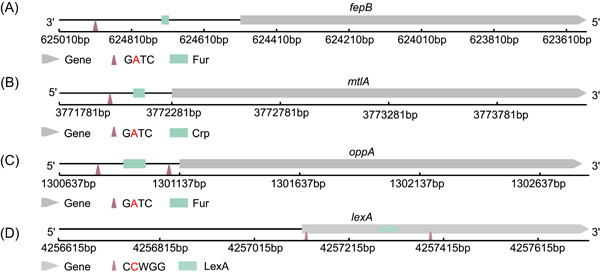
DNA methylation and transcription factor binding site location diagrams based on Bacmethy analysis in *Escherichia coli* K12. (A) Location diagram of GATC motifs (triangles) and Fur binding sites (green rectangle) in *fepB* genes (gray arrow). (B) Location diagram of GATC motifs (triangles) and CRP binding sites (green rectangle) in *mtlA* gene (gray arrow). (C) Location diagram of GATC motifs (triangles) and Fur binding sites (green rectangle) in *oppA* gene (gray arrow). (D) Location diagram of CCWGG motifs (triangles) and LexA binding sites (green rectangle) in *lexA* gene (grey arrow).

To predict whether methylation modifications affect the transcription regulation process, we can scan the vicinity of methylation sites for binding sites or motifs of transcription regulation factors (transcription factor binding site [TFBS]). The presence of DNA methylation and TFBS in close proximity may indicate co‐regulation of gene expression. Taking the Dam motif in *E. coli* K12 as an example, we scanned the RRs of genes containing methylated sites to identify potential TFBS. Using the scanning method, we predicted many co‐regulatory events, some of which have been confirmed in previous studies (Figure 5 and Table S2). In a comprehensive in vivo methylase protection‐based experiment, Tavazoie et al. [17] detected and quantified some TF‐binding sequences in the upstream regions of methylated genes (motif GATC). This included observations for Fur binding to *fepB*, Crp binding to *mtlA*, Crp binding to *flhD*, and Crp binding to *gcd*. Remarkably, our Bacmethy results exhibit similar binding patterns (Figure 5A,B and Table S2). Additionally, our analysis predicted numerous methylation–TFs co‐regulation sites that were previously unknown. As an example, the promoter region of *oppA* contains two GATC motifs, with methylation a fraction of 0.444 and 0.946, respectively. Fur binding sites were found in close proximity to these methylation sites, suggesting co‐regulation (Figure 5C and Table S2). Moreover, we also discovered instances of DNA methylation occurring in the RRs of the TFs themselves. For instance, we detected a C**C**WGG modification, mediated by Dcm, in the promoter region of the LexA operator, which is known to impact the LexA‐mediated SOS response in *E. coli* (Figure 5D and Table S2) [18, 23].

### Building a methylome‐transcriptome integrative regulation model using Bacmethy analysis and RNA‐seq analysis

**Figure 6:**
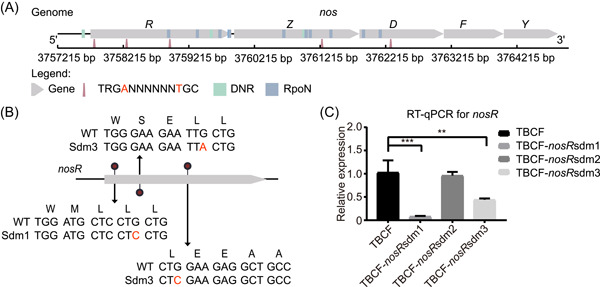
Experimental validation of Bacmethy analysis results for the *Pseudomonas aeruginosa* TBCF strain. (A) Location diagram of TRGANNNNNNTGC motifs (triangles), DNR binding sites (green rectangle), and RpoN binding sites (blue rectangle) in the *nosRZDFYL* operon genes (grey arrow) of *P. aeruginosa* TBCF. Three methylation sites were detected in the *nosR* gene and adjacent DNR and RpoN binding sites were identified. (B) The schematic diagram illustrates the design of point mutation strains (TBCF_*nosR*sdm1, TBCF_*nosR*sdm2, and TBCF_*nosR*sdm3). (C) Quantitative reverse transcription polymerase chain reaction results for TBCF and the three point mutation strains. Each column presents the mean and standard deviation (SD) calculated from three biological replicates per group. **p < 0.01; ***p < 0.001. Comparisons were made using an independent two‐sample *t*‐test.

Previously, SMRT‐seq was used to determine the methylome of a chronically adapted clinical strain of *P. aeruginosa* called TBCF10839. Two methylated motifs (RCC**A**NNNNNNN**T**GAR and TRG**A**NNNNNN**T**GC) recognized by MTases AP and CP were identified [24]. Transcriptomic analysis revealed that the knockout mutant of the *cp* gene (Δ*cp*) significantly reduced the expression of *nosR*, which encodes a nitric oxide reductase transcription regulator. The Δ*cp* mutant showed diminished survival capacity within nitric oxide‐producing RAW 264.7 macrophages and decreased virulence in a *Galleria mellonella* larvae infection model [24]. However, the detailed mechanism by which methylation regulates gene transcription and affects virulence is not fully understood. Using the Bacmethy pipeline, three methylated motifs were detected in the promoters or coding regions of *nosR* gene, along with binding sites for the TF Dnr or sigma factor RpoN (Figure 6A). Among these three methylation sites in *nosR*, the first one was adjacent to DNR binding sites, the third one was adjacent to RopN binding sites, while the second one had no adjacent TF binding sites detected (Figure 6A).

### Experimental verification of the methylome‐transcriptome integrative regulation model

We constructed site‐directed point mutation strains for the three methylation motifs in *nosR* (Figure 6B). TBCF*nosR*sdm1 has a G to C mutation, located 60 bases downstream of the ATG initiation codon of *nosR*; TBCF*nosR*sdm2 has a G to A mutation, located 624 bases downstream of the ATG initiation codon of *nosR*; TBCF*nosR*sdm3 has a G to C mutation, located 1209 bases downstream of the ATG initiation codon of *nosR*. Next, we performed reverse transcription‐quantitative polymerase chain reaction (RT‐qPCR) and found that the expression of the *nosR* gene was significantly downregulated in strains with site‐directed mutagenesis near TF binding sites (sdm1 and sdm3), but not for sdm2 (Figure 6C). This finding suggests that the coordinated regulation of methylation sites and transcriptional regulators may collaboratively control the expression of nitric oxide reductase genes, leading to enhanced anti‐macrophage phagocytosis capacity and virulence.

## DISCUSSION

Bacmethy offers a comprehensive framework for predicting the global impact of bacterial methylation on transcriptional regulation, using PacBio SMRT sequencing data. In the standard Bacmethy analysis, the entire genome is scanned for all possible methylation sites and gene features, while a three‐step workflow was used to classify the methylation sites with potential transcription regulation function. The three steps involve: (i) classification of the DNA methylation level; (ii) Identification of methylation site locations; and (iii) prediction of TF binding.

Bacmethy is a versatile tool. In addition to scanning the promoter regions, Bacmethy is capable of detecting DNA modifications in the coding sequence (CDS) and intergenic regions. This extensive coverage allows for obtaining comprehensive information that goes beyond the promoter regulation. For example, methylation modifications have the potential to impact the curvature of DNA molecules, leading to alterations in gene transcription. Apart from identifying unmethylation (where the modification QV is below the cutoff), Bacmethy also takes into account undermethylation situations, where some sites contain no modification. This observation may imply heterogeneity within the population or a kinetic regulation pattern resulting from the competition of transcriptional factor binding and MTase activity. Furthermore, Bacmethy offers great convenience to users, as it can be accessed through three different methods: command line scripts, docker, and a user‐friendly web tool.

In conjunction with upstream methylome analysis tools (like SMRTLink, REBASE) and downstream functional enrichment software, researchers can achieve a comprehensive mapping of a bacterial strain's methylome and predict potential regulatory functions associated with DNA methylation. Researchers can choose to validate some key regulations derived from Bacmethy analysis through wet lab experiments, such as gene expression quantification and phenotypic analysis of MTase knockout strains under different environmental conditions. The interplay between DNA methylation and transcriptional factor binding can be experimentally verified using site‐directed mutagenesis, RT‐qPCR analyses, electrophoretic mobility‐shift assay, among others.

Through the utilization of benchmark data sets and our own data, we demonstrate the significant contribution of Bacmethy in understanding the distinct characteristics of DNA methylation modifications and accurately predicting their impact on transcriptional regulation. In *E. coli* K12, our method successfully predicted numerous co‐regulation events, with validations available from previous studies for some of them. Additionally, our method also revealed novel mechanisms that suggest previously unknown regulatory relationships. Besides, by employing Bacmethy and transcriptome data of a *P. aeruginosa* DNA MTase knockout strain and a control strain, we raise a model for carrying out bacterial methylome‐transcriptome integrative regulatory analysis. We predicted and experimentally validated that DNA methylation sites and TFs cooperatively regulate the expression of certain NO reductase‐related gene, which provides an explanation for the strain's remarkable antiphagocytosis ability.

## CONCLUSION

There has been an explosion of bacterial complete genome and epigenome investigation in the last decades, aided by the expanded application of real‐time sequencing technologies. However, the lack of analysis tools has impeded the comprehensive annotation of these epigenomes. The Bacmethy tool we developed can contribute to the identification of putative novel targets of DNA methylation regulation, thereby deepening our understanding of bacterial epigenetics network and advancing our knowledge of cellular and physiological function regulation.

## METHODS

### Bacmethy pipeline

The Bacmethy pipeline consists of the following steps (Figures 1 and S1): 

#### Prerequisite step

SMRT‐seq data can be generated in‐house or downloaded from the public database. It is worth noting that a significantly higher sequencing coverage is required to detect m5C modifications compared to m6A and m4C modifications using SMRT‐seq technology. The current suggested minimum coverage per strand is 25× for m6A and m4C, and 250× for m5C. The required coverage should aim at 250× for detection of unknown modifications [25].

The Bacmethy pipeline requires three input files (genome.fa, motifs.gff, and motifs.csv files). Typically, you can obtain the files from a public database or through the SMRT‐seq facility. The Pacbio SMRTseq facility is equipped with the preinstalled SMRTLink software. Nevertheless, if you need to carry out SMRTLink analysis on your own, you may download the tool and follow the instructions (https://pacbio.cn/support/software-downloads/). The tool includes the ability to assemble genomes using “Microbial Assembly Application” and detect base modifications while constructing motif‐specific models through “Base Modification Analysis Application.” When using the “Base Modification Analysis Application, it is important to add the fraction and motif requirements (‐t kineticstools_compute_methyl_fraction = true ‐t run_find_motifs = true) to obtain methylation motif and methylation fraction information accurately. The output files (genome fasta file, Motifs.csv and Motifs.gff) will be used for Bacmethy analysis.

#### Step 1: Gene annotation and location

The assembled genome (fasta file) generated from SMRTLink can be annotated using tools like PROKKA v1.14.6 (‐‐species ‐‐metagenome ‐‐Kingdom bacterium) [26]. The pipeline then assigns regulatory, CDS, and intergenic regions for each gene. RR was set by default from 500 bp upstream of the ATG initiation codon to 100 bp downstream of the ATG. In cases where the 500 bp upstream region overlaps with the upstream gene in the same strand, the start site of the RR will be automatically set to the base immediately after the end of the upstream gene. The output files in this step are the region location files, which are saved as bed files (*region.bed).

#### Step 2: Methylation sites classification

The “Motifs.csv” and “Motifs.gff” files generated by the SMRTLink tool were utilized as inputs for Bacmethy. By default, Bacmethy searches for all three types of bacterial genome methylation (m6A, m4C, m5C). Several parameters are important for methylation site classification, including modification QV, methylation fraction (methylated–read ratio), and reads coverage (the coverage level of the reads used to make the call). The default cut‐off modification QV score of 40 corresponds to a methylation probability of 0.9999. While the methylation fraction (methylated–read ratio) represents the percentage of modified reads within the total reads (modified reads + unmodified reads) with a motif site [27]. For example, the methylation fraction of 75% means that for the methylated site, 75% reads containing the methylated base, and the remaining 25% contain no methylation. The read coverage is another important parameter, as data with insufficient coverage cannot be deemed reliable. In our analysis, the default coverage cut‐off is set to 30, meaning that only motifs with a coverage of 30 or higher will be taken into account. Using the metrics mentioned above, methylation motif sites can be classified as unmethylated, methylated, or undermethylated. Undermethylation is considered an intermediate stage, where motifs are detected as methylated, but the methylation fraction is notably low. Users can adjust the metrics for undermethylated and unmethylated motifs identification as needed. The default unmethylated cutoff is set to “modification QV < 40” for each motif site. The default methylated cutoff is set to “modification QV ≥ 40 and methylation fraction ≥ 0.75” for each motif site. The default undermethylated cutoff is “modification QV ≥ 40 and methylation fraction < 0.75.” The output files for this process are tables summarizing the location of methylation sites (Target_methylation.bed, Target_unmethylation.bed, and Target_undermethylation.bed). Besides, the pipeline generates methylation ratio plots (e.g., Figure 2A), which show the distribution of the methylation fractions of the motifs in the bacterial stains. The pipeline additionally produces DNA methylation distribution plots (e.g., Figure 2B), which show the identification QV, read coverage, and methylation fraction. These plots are generated using R (R1: Methylation distribution plots).

#### Step 3: Filtering candidate genes and regions with methylation motifs

In this step, the “Target_methylation.bed” and “Regulation_Region.bed” files serve as input. BEDTools' intersect function is employed to detect overlapping regions between the methylation modification locations and the regulation regions, indicating where methylation events have occurred in these regions. Additionally, text mining is utilized to match “Regulation Region” and “Gene,” which is then added to the result summary. The output files are tables that summarize information about methylated genes (Figure S1; R2: Methylated_region.fasta and Methylated_geneset_table). In addition to regulatory regions, the pipeline can also be used for the examination of DNA modifications across CDS and intergenic regions. Importantly, our pipeline can also produce the locations of unmethylated or undermethylated motifs. This expanded coverage can provide a more comprehensive picture of the mechanisms of transcriptional regulation.

#### Step 4: Transcription regulation effector binding motif scanning

Position‐dependent letter probability matrices of each TF (TF, including sigma factor) binding motif are needed at this step. Users can download position frequency matrices (PFMs) from databases such as CollecTF [28]. Our pipeline also provides a module that converts PFMs to position probability matrices. Next, the FIMO tool, with default parameters (‐oc ‐verbosity 1 ‐thresh 1.0E‐4 PMM.matrix methylated_region.fasta), is used to scan each RR with DNA methylation to find regions containing TFBS. Finally, a shell script is utilized to identify genes that are potentially regulated by both DNA methylation and TFs (RTMG: Regulated by transcriptional factor and methylation gene)(output files: RTMG region.fasta and RTMG list).

#### Optional step: Circos plot of methylome

The methylation distribution at the genome level can be visualized using Circos [29]. Bacmethy generates all Circos configuration files using a self‐script in bash. The outer ring represents the assembled genome, while the next two rings represent the genes encoded on the positive and negative strands, respectively. The fourth and sixth circles show the methylated motifs on the positive and negative strands, respectively, where the connection between two rings denotes a region of undermethylation and unmethylation motifs on the genome. Finally, the inner ring illustrates the distribution of whole DNA methylation motifs across the genome.

#### Optional step: Correlation with transcriptomics data

Whole‐genome methylation can be abolished by MTase knockout in a given strain. By performing MTase knockout in strains and pairing this with RNA‐seq analysis, users can identify differentially expressed genes (DEGs) between the altered strain and the wild‐type strain, isolating potential genes regulated by DNA methylation. Importantly, overlap between DEGs and RTMGs can serve as convincing evidence for DNA methylation‐mediated gene regulation. Furthermore, researchers can focus on the overlap list, particularly within gene families that are often closely spaced and play a similar role.

#### Optional step: Function enrichment

Users can perform function enrichment using the gene lists generated in the above steps. COG category [20] annotation and DAVID [22] tools were used. BlastP and similar alignment tools were employed for protein function annotation. The results were visualized using the R package ggplot2 [19].

### Docker and web server construction

Bacmethy employs Docker (https://www.docker.com/), a container platform to build, manage, and run applications from macOS, Linux, or Windows (Figure 1). To run Bacmethy, users can download the Docker Bacmethy images from the Docker hub (https://hub.docker.com/repository/docker/liujihong/bacmethy) using the Docker pull command.

Bacmethy was also made as a user‐friendly web server interface available online. VUE 2.6.14 and KOA 2.7.0 were used in the website development process.

Users can visit https://bacmethy.med.sustech.edu.cn to access the server and must provide DNA methylation description files (motifs.csv and motifs.gff) obtained from SMRT sequencing, as well as the complete genome file in FASTA format (Figure S2). Upon completion of the Bacmethy program, users will receive an email containing the Bacmethy result.

### Bacterial strains and data sets

This study includes the methylome data of 13 DNA MTases from seven bacterial strains (Table 1). Each MTase recognizes a different motif, such as G**A** TC, TRG**A**NNNNNNTGC, and C**C**WGG. The methylated nucleotide within the motif is highlighted in bold. The underlined letter denotes the nucleotide that pairs with the methylated nucleotide on the complementary strand. The methylome data were generated from SMRT‐seq data. SMRT‐seq data of four bacterial strains, including four MTases from *E. coli* MG1655, two MTases from *S. aureus* USA300, M.Xsp91I (*Xanthobacter* species 91), or M.CmaLM2III (*Clostridium mangenotii* LM2), respectively, were obtained from the GEO database using accession number GSE69872 [4]. Additionally, we incorporated SMRT‐seq data of three in‐house strains, *P. aeruginosa* TBCF, LYSZa2, and LYSZa7. The genomes of *P. aeruginosa* LYSZa2, LYSZa7, and TBCF were sequenced using SMRT‐seq and analyzed by our team, following the method described in our previous publication [19, 24]. In brief, *P. aeruginosa* strains were cultured in Luria‐Bertani (LB) broth, genomic DNA was extracted, and SMRT sequencing was performed. PacBio reads were assembled into complete genomes using the HGAP4 pipeline of SMRTLink software v9.0 with default settings, and the complete genomic sequences were used as the reference sequences. SMRTLink's Base Modification Analysis and Motif Analysis applications were used to perform DNA methylation analysis. The output files generated (motids.gff and motifs.csv) were used for Bacmethy analysis. The REBASE database was used to predict and assign recognition motifs to the identified MTase genes, including those impacting the restriction modification system. Furthermore, we leveraged RNA‐seq data sets from *P. aeruginosa* TBCF and TBCF with MTase gene knockout [24] to establish a correlation with the Bacmethy analysis outcomes.

### Construction of site‐directed mutagenesis *P. aeruginosa* TBCF strains

Strains and plasmids used in this part of the study are listed in Table 2. All primers are listed in Table S3. *E. coli*, *P. aeruginosa* TBCF, and its mutant strains were routinely grown in LB liquid medium or on LB agar plates containing 1.5% (w/v) agar at 37°C overnight. Gentamicin (60 μg/mL) and triclosan (25 μg/mL) were used to screen *E. coli* and mutant strains of *P. aeruginosa*.

**Table 2:** Bacterial strains and plasmids used in this study.

Name	Description	Reference or source
*Pseudomonas aeruginosa*		
TBCF10839	CF isolate	[30]
TBCF_*nosR*sdm1	The first site‐directed mutation on *nosR* of TBCF (base G mutated to C, located 60 bases downstream of the initiation codon ATG of *nosR*)	This study
TBCF_*nosR*sdm2	The second site‐directed mutation on *nosR* of TBCF (base G mutated to A, located 624 bases downstream of the initiation codon ATG of *nosR*)	This study
TBCF_*nosR*sdm3	The third site‐directed mutation on *nosR* of TBCF (base G mutated to C, located 1209 bases downstream of the initiation codon ATG of *nosR*)	This study
*Escherichia coli*		
DH5a	cloning strain; dam^+^ dcm^+^	TransGen

Site‐directed mutant strains were created using a two‐step allelic exchange method [31]. Basically, the mutation on a specific gene was achieved by amplifying upstream and downstream DNA fragments that contained the single‐nucleotide mutation using two primer pairs (Table S3; primer lists SDM1–SDM3). The resultant PCR products were purified with the HiPure PCR Purification Kit (Magen), and then ligated onto the *Hin*dIII‐ and *Bam*HI‐digested pK18mobSacB vector via the Seamless Assembly Cloning Kit (Clone Smarter). The ligation products were then transformed into *E. coli* DH5α and screened for positive clones with gentamicin. *E. coli* with the designated site‐directed mutagenesis fragments was identified by Sangon PCR sequencing. The plasmids of *E. coli* DH5α were then conjugated into the *P. aeruginosa* TBCF with the help of RK600 strain. Monoclonal colonies were screened for gentamicin and triclosan resistance, and selected colonies were grown on NaCl‐free LB agar plates with 20% sucrose. Finally, colonies without gentamicin resistance were selected, and PCR and Sangon sequencing was utilized to confirm the site‐directed mutagenesis strains.

### RNA extraction and reverse transcription‐quantitative PCR

*P. aeruginosa* TBCF and its mutant strains were grown in LB liquid medium overnight at 37°C, 200 rpm. On the following day, overnight cultures were inoculated into fresh LB at 1% and grown to OD600 = 1.0. The bacteria were then collected by centrifugation at 12,000 rpm for 3 min. The Eastep Super Total RNA Extraction Kit (Promega) was utilized to extract total RNAs from the strains. Residual genomic DNA (gDNA) was removed and complementary DNA (cDNA) was synthesized from 1 μg of DNA‐free RNA with the Evo M‐MLV RT Kit with gDNA Clean for qPCR (Accurate Biology). The SYBR® Green Premix Pro Taq HS qPCR Kit (Accurate Biology) and qPCR systems (Roche) were used to perform RT‐qPCR assays as per the manufacturer's instructions. The negative control group replaced the cDNA template with nuclease‐free water. Samples were set up on a LightCycler 480 Multiwell Plate 96, covered with a clear sealing cover (Roche), and RT‐qPCR primers were listed in Table S3. The LightCycler® Software (Roche) was employed to analyze the data. The LightCycler 96 (Roche) was used in the following PCR protocol: one cycle at 95°C for 30 s (None mode), followed by 40 cycles at 95°C for 5 s (None mode), 60°C for 30 s (Single mode), and one cycle at 95°C for 10 s (None mode), 65°C for 60 s (None mode), and 97°C for 1 s (Continuous mode). By normalizing against the expression of housekeeping gene rplU, the relative expression levels of target genes in TBCF and mutants were calculated and compared. All experiments were conducted with at least three biological replicates. Comparisons were made using an independent two‐sample *t*‐test. Statistical parameters are included in the figure legends.

## AUTHOR CONTRIBUTIONS

Yang Liu and Liang Yang conceived the idea, supervised the project, and edited the manuscript. Ji‐Hong Liu led software development, data curation, and formal analysis. Yizhou Zhang tested and amended the codes and performed wet lab experiments. Ji‐Hong Liu and Yizhou Zhang drew pictures. Yizhou Zhang, Ning Zhou, and Jiale He provided validation. Ji‐Hong Liu, Zhao Cai, and Yang Liu drafted the original manuscript. Yang Liu, Liang Yang, and Ning Zhou were responsible for funding acquisition. Cai Zhao, Ning Zhou, and Jing Xu participated in writing review and editing. All authors have read the final manuscript and approved it for publication.

## CONFLICT OF INTEREST STATEMENT

Yang Liu, Liang Yang, and Ji‐Hong Liu are listed as inventors on a patent application related to the analysis tool and system used in this study. This patent application is currently undergoing substantive review (application number 202210809556.X, China National Intellectual Property Administration). The remaining authors declare no conflict of interest.

## Supporting information


**Figure S1**: Bacmethy pipeline workflow.
**Figure S2**: Screenshot of the Bacmethy website submission page.
**Figure S3**: The distribution of methylation fraction and sequencing quality.
**Figure S4**: Circos plots of methylome.

**Table S1**: Distribution of methylated or un(der)methylated MTase recognition sites in bacteria genomes. MTase, methyltransferase.
**Table S2**: Example of co‐location of methylated (or undermethylated) MTase recognition sites and TF binding sites in *Escherichia coli* K12. TF, transcription factors.
**Table S3**: Primers used for mutant strain construction and RT‐qPCR.

## Data Availability

Data sets of *Pseudomonas aeruginosa* LYSZa7, LYSZa2, TBCF, and TBCF mutant are available at NCBI SRA, with accession numbers PRJNA656096 (https://www.ncbi.nlm.nih.gov/bioproject/?term=656096), PRJNA918318 (https://www.ncbi.nlm.nih.gov/bioproject/?term=918318), PRJNA830320 (https://www.ncbi.nlm.nih.gov/bioproject/?term=830320), and PRJNA835892 (https://www.ncbi.nlm.nih.gov/bioproject/?term=835892), respectively. An open‐source package of Bacmethy is available on GitHub (https://github.com/LiuJih2021/Bacmethy). All the scripts and data are also available on GitHub (https://github.com/LiuJih2021/bacmethyFigures) and Figshare (https://figshare.com/s/b216fe9272deb7e6caa8). Supplementary Materials (figures, tables, scripts, graphical abstract, slides, videos, and update materials) may be found in the online DOI or iMet a Science (http://www.imeta.science/).
