# Supplementary material for: Bacmethy: A novel and convenient tool for investigating bacterial DNA methylation pattern and their transcriptional regulation effects
Source: Imeta. 2024 Mar 19;3(3):e186. doi: 10.1002/imt2.186 (PMC11183182; doi:10.1002/imt2.186)
Supplement: Supplementary file 1 — Figure S1: Bacmethy pipeline workflow. Figure S2: Screenshot of the Bacmethy website submission page. Figure S3: The distribution of methylation fraction and sequencing quality. Figure S4: Circos plots of methylome. [file IMT2-3-e186-s002.docx]

**Supporting information to:**

**Bacmethy: a novel and convenient tool for investigating bacterial DNA methylation pattern and their transcriptional regulation effects**

**Running title:** pipeline for studying bacterial transcriptional regulation by DNA methylation

Ji-Hong Liu^1,2^, Yizhou Zhang^1,2^, Ning Zhou^3^, Jiale He^1,2^, Jing Xu^1^, Zhao Cai^2^, Liang Yang^2,4^*, Yang Liu^1^*

^1^Medical Research Center, Southern University of Science and Technology Hospital, Shenzhen 518055, China

^2^School of Medicine, Key University Laboratory of Metabolism and Health of Guangdong, Southern University of Science and Technology, Shenzhen 518055, China

^3^Clinical Laboratory, Southern University of Science and Technology Hospital, Shenzhen 518055, China

^4^Shenzhen Third People’s Hospital, The Second Affiliated Hospital of Southern University of Science and Technology, National Clinical Research Center for Infectious Disease, Shenzhen 518112, China

*Correspondence: liuy7@sustech.edu.cn (Yang Liu), yangl@sustech.edu.cn (Liang Yang)

**Supplementary Figures**


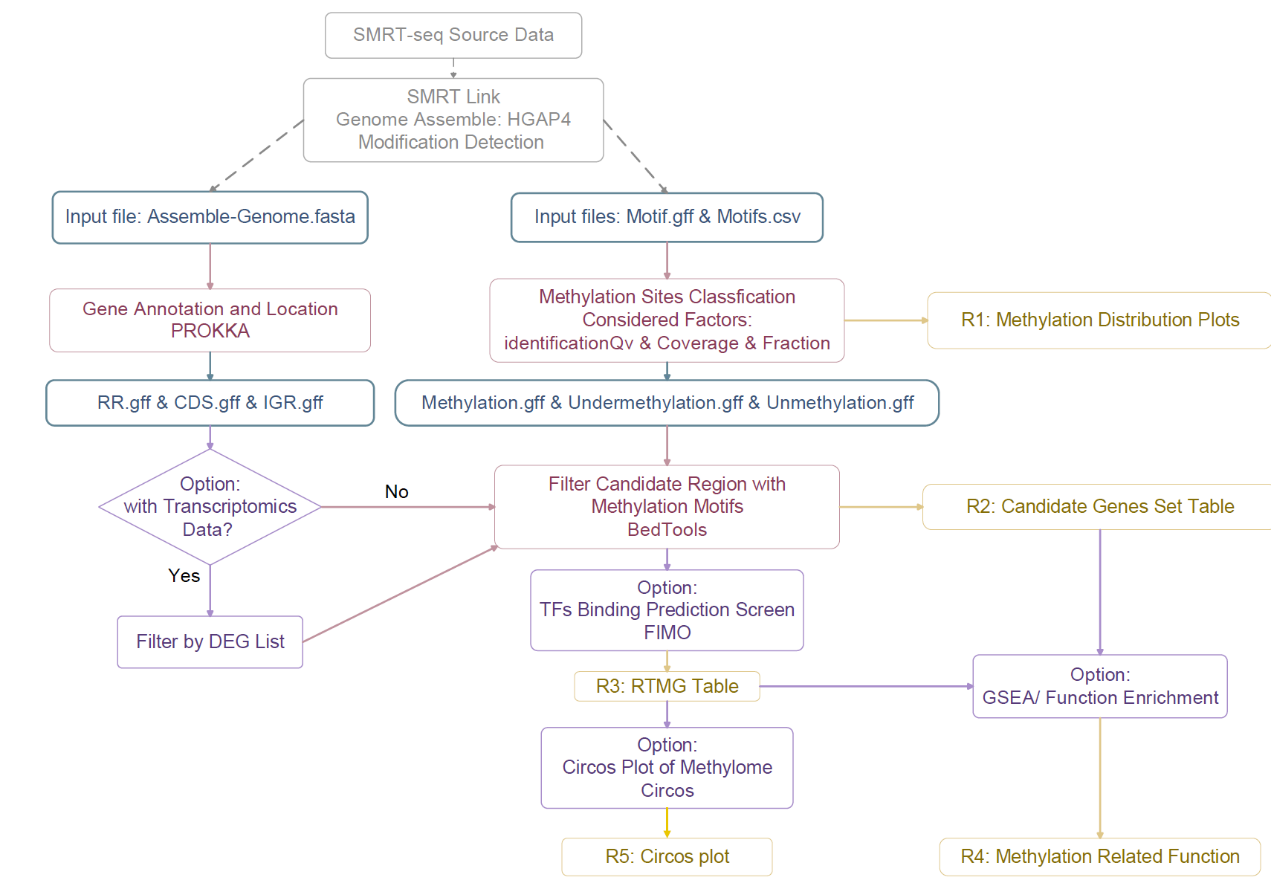


**Figure S1 Bacmethy pipeline workflow.** The Bacmethy pipeline consists of several steps. Prerequisite Step. SMRTLink tool can be used to assemble genome, detect base modification, and construct motif-specific models. The files generated can be used for Bacmethy pipeline. Step 1. Gene annotation and localization. Using PROKKA [1] to perform genome annotation and extract regulation region. Step 2. DNA Methylation sites classification. Differential methylation type and methylation state (methylation, unmethylation or undermethylation) will be divided into different analyses. Step 3. Filtration of candidate gene and regulation with methylation motifs. Genes with different methylated status in regulation regions were selected for downstream analysis. Option Step 1. TF binding prediction screen. FIMO was used to scan the regulation region with DNA methylation and to find potential TF binding position, and output the potential genes regulated by both DNA methylation and TFs (RTMG table and list). Option Step 2. Correlation with transcriptomics data. Filter the overlap of DEG which is wild-type strain and methyltransferase knockout strain and RTMG in BMITP. Option Step 3. GSEA/Function Enrichment. Find potential function of above gene lists.


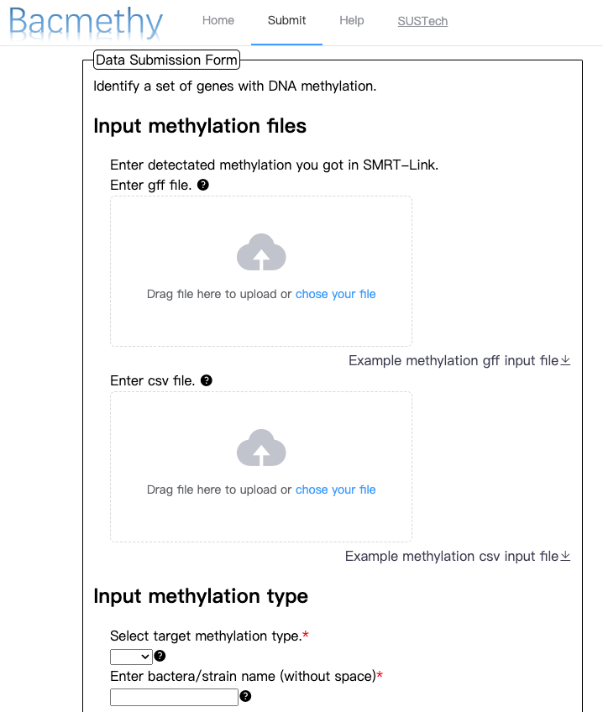

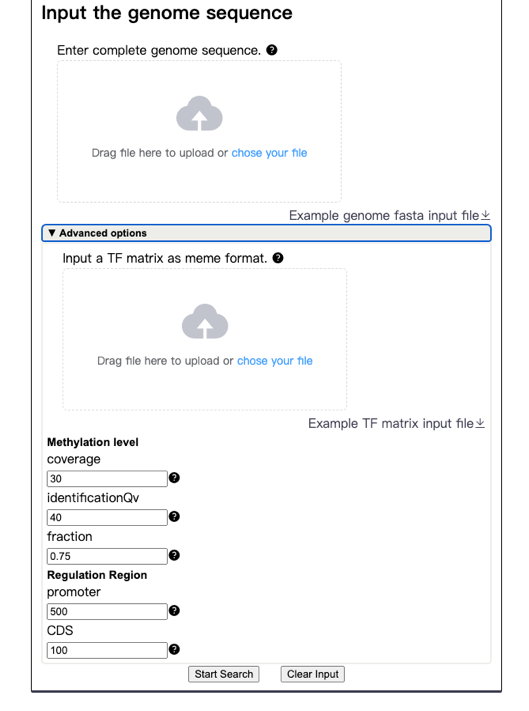


**Figure S2 Screenshot of the Bacmethy website submission page.**

**Figure S3** The distribution of methylation fraction and sequencing quality. (A) The distribution of methylation fraction per motif in the bacterial stains, where the title of each circle includes the strain name and recognition motif, and the counts indicate the number of motifs with diverse ratios of 0 - 60% (red), 60 - 75% (grey), 75 - 90% (blue), and 90 - 100% (green). The scatter plots for the quality distribution of methylated and un(der)methylated motifs across the strains per MTase motif (B) for the five strains (For *Xanthobacter sp. 91* and *Clostridioides mangenotii LM2*, only the orphan MTases were show). The x-axis shows the reads coverage, and the y-axis shows the identification QV. The methylation fraction is represented by dot color, with blue indicating a high methylation fraction and red indicating a low fraction.

**Figure S4 Circos plots of methylome. Circos plot of MTase methylated motifs in the chromosomes of 9 bacterial strains.** The outer ring is the genome range. The next two inner rings represent the genes encoded on the positive and negative strands respectively. Next, the fourth and sixth circle represents motifs with methylation on the positive and negative strand respectively; the loop between the two represents motifs with undermethylation and unmethylation on the genome. The inner ring indicates the distribution of all MTase recognized motifs on the genome.

**References:**

1. Seemann T. 2014. “Prokka: rapid prokaryotic genome annotation.” *Bioinformatics* 30: 2068-2069. <https://doi.org/10.1093/bioinformatics/btu153>
